# Internet Search and Krokodil in the Russian Federation: An Infoveillance Study

**DOI:** 10.2196/jmir.3203

**Published:** 2014-09-18

**Authors:** Andrey Zheluk, Casey Quinn, Peter Meylakhs

**Affiliations:** ^1^Menzies Centre for Health PolicyUniversity of SydneyUniversity of SydneyAustralia; ^2^PRMA Consulting LtdFleet, HampshireUnited Kingdom; ^3^ Laboratory for Internet StudiesNational Research University, Higher School of EconomicsSt PetersburgRussian Federation

**Keywords:** Russia, search engine, surveillance, controlled substances, designer drugs, street drugs

## Abstract

**Background:**

Krokodil is an informal term for a cheap injectable illicit drug domestically prepared from codeine-containing medication (CCM). The method of krokodil preparation may produce desomorphine as well as toxic reactants that cause extensive tissue necrosis. The first confirmed report of krokodil use in Russia took place in 2004. In 2012, reports of krokodil-related injection injuries began to appear beyond Russia in Western Europe and the United States.

**Objective:**

This exploratory study had two main objectives: (1) to determine if Internet search patterns could detect regularities in behavioral responses to Russian CCM policy at the population level, and (2) to determine if complementary data sources could explain the regularities we observed.

**Methods:**

First, we obtained krokodil-related search pattern data for each Russia subregion (oblast) between 2011 and 2012. Second, we analyzed several complementary data sources included krokodil-related court cases, and related search terms on both Google and Yandex to evaluate the characteristics of terms accompanying krokodil-related search queries.

**Results:**

In the 6 months preceding CCM sales restrictions, 21 of Russia's 83 oblasts had search rates higher than the national average (mean) of 16.67 searches per 100,000 population for terms associated with krokodil. In the 6 months following restrictions, mean national searches dropped to 9.65 per 100,000. Further, the number of oblasts recording a higher than average search rate dropped from 30 to 16. Second, we found krokodil-related court appearances were moderately positively correlated (Spearman correlation=.506, *P*≤.001) with behaviors consistent with an interest in the production and use of krokodil across Russia. Finally, Google Trends and Google and Yandex related terms suggested consistent public interest in the production and use of krokodil as well as for CCM as analgesic medication during the date range covered by this study.

**Conclusions:**

Illicit drug use data are generally regarded as difficult to obtain through traditional survey methods. Our analysis suggests it is plausible that Yandex search behavior served as a proxy for patterns of krokodil production and use during the date range we investigated. More generally, this study demonstrates the application of novel methods recently used by policy makers to both monitor illicit drug use and influence drug policy decision making.

## Introduction

### Overview

Krokodil, otherwise known as desomorphine, is a cheap injectable drug easily synthesized in household kitchens from codeine-containing medication (CCM). The first confirmed report of krokodil use in Russia occurred in 2004. In 2012, reports of horrific krokodil-related injection injuries began to appear beyond Russia in Western Europe [1] and the United States [2]. We conducted this exploratory study to determine if several complementary data sources may provide insight into the relative scale and spatial patterns of behaviors consistent with an interest in the production and use of krokodil before and after the imposition of Russian federal restrictions on CCM sales in 2012.

### Review of Literature on Krokodil

Current scientific literature on krokodil is limited. We reviewed international literature available through PubMed and Google Scholar. In addition, we searched the four most popular Russian online news sources [3] for the term “desomorphine” in the date range January 2009 to December 2012 using Yandex News (see Table 1). Across the four sources, we identified 929 Russian language articles associated with the term “desomorphine” in this date range, which was bounded by a period of increasing public interest in 2009, and the 6-month period following federal restrictions on CCM sales across Russia in June 2012.

**Table 1:** Russian news sources reviewed via Yandex News (Jan 1, 2009 to Dec 31, 2012).

Source	Website	Count (N=929)	Orientation
RIA Novosti	rian.ru	103	State-owned
Vesti.ru (Website of Russia 24 TV)	vesti.ru	38	State-owned
Komsomlskaya Pravda	kp.ru	748	Private, tabloid
Russia business consulting	rbk.ru	40	Private, business focus

### The Origins of Krokodil

Current literature describing the origins of krokodil in Russia is vague. Time magazine reported the first appearance of krokodil in the Siberian and the Far East Federal Regions of Russia in the early 2000s [4]. We identified the first Russian news report of krokodil use in the Komi Republic in the western part of the Siberian Federal Region in May 2004 [5]. A police report from 2004 described the seizure of a new illicit drug never before seen in Russia called desomorphine. Conversely, a 2010 video produced by the Russian Drug Control Service (FSKN) suggested krokodil first appeared in the Komi Republic in 2002 [6] and that by 2006, 19 Russian oblasts (subregional administrative units) were affected. These affected oblasts were primarily in the Siberian, Volga, Northwestern, and Central Federal Regions. From 2006 onwards, krokodil use increased dramatically according to Russian news reports [7]. The ease of access to low-cost CCM and ease of domestic manufacture were widely reported as contributing to the spread of krokodil use [1]. Shortages of heroin during 2010 have been described as a further factor contributing to krokodil use. Several authors suggested krokodil largely displaced traditional opiates as a consequence of Afghan heroin shortages after 2010 [1,4,8,9]. A 2012 police report stated seizures of krokodil grew by 40 times from 2 kg in 2006 to 100 kg in 2011. By comparison, a mean of 2922 kg of heroin were seized each year between 2006 and 2010 in the Russian Federation [10,11].

### Prevalence of Krokodil Use

Estimates of the scale of krokodil use diverged markedly. In 2011, a senior Russian addiction medicine specialist reported 5000 krokodil users were receiving treatment nationally, out of a total estimated national population of 20,000-30,000 users [12]. In June 2012, the FSKN estimated between 5000 and 7000 deaths were attributable to krokodil in Russia over the preceding 2 years [7]. Also in 2012, international researchers estimated 100,000 people were krokodil dependent in Russia, while suggesting the actual number could be higher [13]. Pharmacy sales of CCM provided a further indicator of the scale and spatial distribution of krokodil use. For example, in the Urals Region, the FSKN reported an increase in annual CCM sales from 4.2 million in 2007 to 12 million packets in 2010 [14].

There were limited data describing the spatial distribution of krokodil use before the federal restrictions on sales of CCM in June 2012. Krokodil use had been widely reported across Russia and bordering Ukrainian regions [15,16]. A December 2011 news report citing “various sources” described the results of toxicology tests conducted in several Russian oblasts [17]. According to this unreferenced news report, toxicology tests suggested 20% of people who inject drugs (PWID) in Chechnya were krokodil users, compared with 60% in Kazan and Ryazan, and 90% in Yaroslavl oblast. Overall, the publicly available data on the scale and prevalence were both limited and fragmented [18] before the introduction of restrictions in June 2012.

### Researching Krokodil

A series of articles reviewing the current scientific understanding of krokodil appeared in the International Journal of Drug Policy in 2013. One of these articles by Grund et al consolidated current scientific information, including interview data from Russian key informants [13]. The authors pointed to the paucity of scientific research into krokodil prevalence and use. Several commentary articles accompanied Grund et al’s review. In one accompanying commentary piece, Heimer described the difficulties of investigating the prevalence of krokodil use without ethnographic and epidemiological data [18]. Heimer further noted the time lag between submitting research proposals and fieldwork, describing how his personal efforts to set up field interviews were thwarted by changes in krokodil use patterns resulting from federal restrictions on the sale of CCM in June 2012. In summary, conducting research into krokodil was more complex than with many established illicit drugs because of the novel nature of the substance and policy changes.

### Morbidity and Mortality Associated With Krokodil in Russia

Desomorphine was originally developed as a morphine substitute. It was first synthesized in the United States in 1932, with the aim of producing a low-cost substitute with minimal side effects [19]. However, laboratory-synthesized desomorphine was regarded as an unsuccessful substitute, being shorter lasting, stronger, and more addictive than morphine. In Russia, krokodil, or what is termed desomorphine, is an illicit injectable drug domestically manufactured from codeine, iodine, phosphorus, paint thinner, and lighter fuel [13]. The resulting substance is regarded as impure, creating the potential for severe injury among PWID. It is the presence of impurities that has produced consistent reports of injuries characteristic of krokodil use. Characteristic injuries have included vascular damage, skin and soft tissue infections, necrosis and gangrene [20,21], as well as burns associated with domestic manufacture [22]. The short duration of narcotic effects, strong dependence, and chemical instability of the domestically produced drug has led to reports of binges of frequent injecting among krokodil users [23].

Frequent injecting is generally regarded as a risk factor for human immunodeficiency virus (HIV) and other injecting-related harms [24,25]. Further, PWID experience multiple comorbidities, creating a disproportionate need for health services [13,26]. However, as a consequence of punitive drug laws, Russian PWID are often particularly unwilling to seek medical assistance, exacerbating injecting-related injuries [27-29]. In summary, the characteristics of drug preparation and use, as well as the legal environment in which PWID use illicit drugs increased the morbidity and mortality associated with krokodil use in Russia before the restrictions on CCM in 2012.

### Russian Policy Responses to Krokodil

The easing of restrictions on access to CCM may have increased the production and use of krokodil in Russia. During the Soviet period up to 1991, CCM was available only through pharmacies with a medical prescription [2]. Following the fall of USSR in 1991, restrictions on sales of CCM were removed. In 2004, Russian manufacturers introduced new CCMs targeted at Russian consumers. Following advocacy by the largest Russian manufacturer, Pharmstandard, these new CCMs remained accessible to consumers without medical prescriptions [30]. While a two-packet-per-person limit on CCM sales formally existed, this was routinely ignored by pharmacists [31]. From the mid-2000s, Russian government agencies explicitly linked the unrestricted access to CCM with the illicit consumption of krokodil [32]. After 2009, krokodil became increasingly recognized as a public health and policy problem. Increasing public concern was accompanied by Russian media reports of conflicts of interest. In particular, the relationship between Pharmstandard and the Russian Minister of Health became the target of media scrutiny [33,34]. Media reports suggested Pharmstandard’s advocacy had prevented the imposition of restrictions on the sale of CCM. In summary, several failures in CCM regulation may have facilitated the expansion of krokodil use in Russia.

### National Restrictions on Codeine-Containing Medication

At a national drug control conference in 2011, Russian President Medvedev announced restrictions on the sale of CCM without medical prescriptions [35]. Federal restrictions on CCM sales were original scheduled to start mid-2011. However, the Russian Ministry of Health effectively delayed the implementation of restrictions for 12 months. Ministry of Health officials argued that 40 million individuals using CCM for intended analgesic purposes would be disadvantaged by premature restrictions [36]. In opposing immediate restrictions, the Ministry of Health also drew on widespread public opposition to restrictions on CCM sales [37] (see Table 2). The delays in restrictions led to conflict in Russian national media between the FSKN, academics [38], and the Ministry of Health [36]. The public debate over CCM restrictions thus illuminated interagency tensions in an otherwise generally opaque Russian federal health policy landscape.

**Table 2:** Public opinion survey into consequences of proposed federal CCM restrictions May 2011 [34].

	How will this affect the battle against drugs in Russia?	How will this affect the needs of ordinary patients?
Generally positive	32%	11%
No effect	49%	21%
Generally negative	5%	56%
Difficult to answer	14%	12%

### The Effect of Restrictions on Codeine-Containing Medication

From 2011 to June 2012, several Russian oblast governments implemented interim local restrictions on CCM sales [39,40]. These interim restrictions were directed at reducing the production and consumption of krokodil in advance of federal bans [41,42]. On June 1, 2012, a federal law restricted CCM sales across all of Russia. Russian media subsequently reported decreased sales volumes of CCM [43]. However, media reports of krokodil production and use and krokodil-related arrests continued [44,45]. These media reports suggested the federal restrictions on CCM sales had been only partially effective in curtailing krokodil production and use.

### Krokodil and the Internet

Between 2010 and 2012, Russian policy makers emphasized the negative influence of the Internet in disseminating krokodil-related information. In April 2011, the FSKN presented the results of its research into Internet search patterns for krokodil-associated terms [46]. The FSKN described a marked increase in Internet searches for methods of preparing and purchasing krokodil, from 3000 searches during 2010, to 50,000 in the first 3 months of 2011. Several days later, Russian President Dmitry Medvedev reiterated Russian government concerns about the relationship between krokodil and the Internet at a national forum dedicated to illicit drug use prevention. President Medvedev demonstrated that when the term “desomorphine” was entered into the Yandex search engine, the first results revealed information on how to prepare the drug [35]. Medvedev suggested these results proved that Internet users were most interested in producing desomorphine, rather than simply searching for general information about the drug. This widely reported demonstration by the Russian President served as the catalyst for subsequent federal restrictions on CCM.

### Russian Government Statements About Krokodil

**Figure 1:**
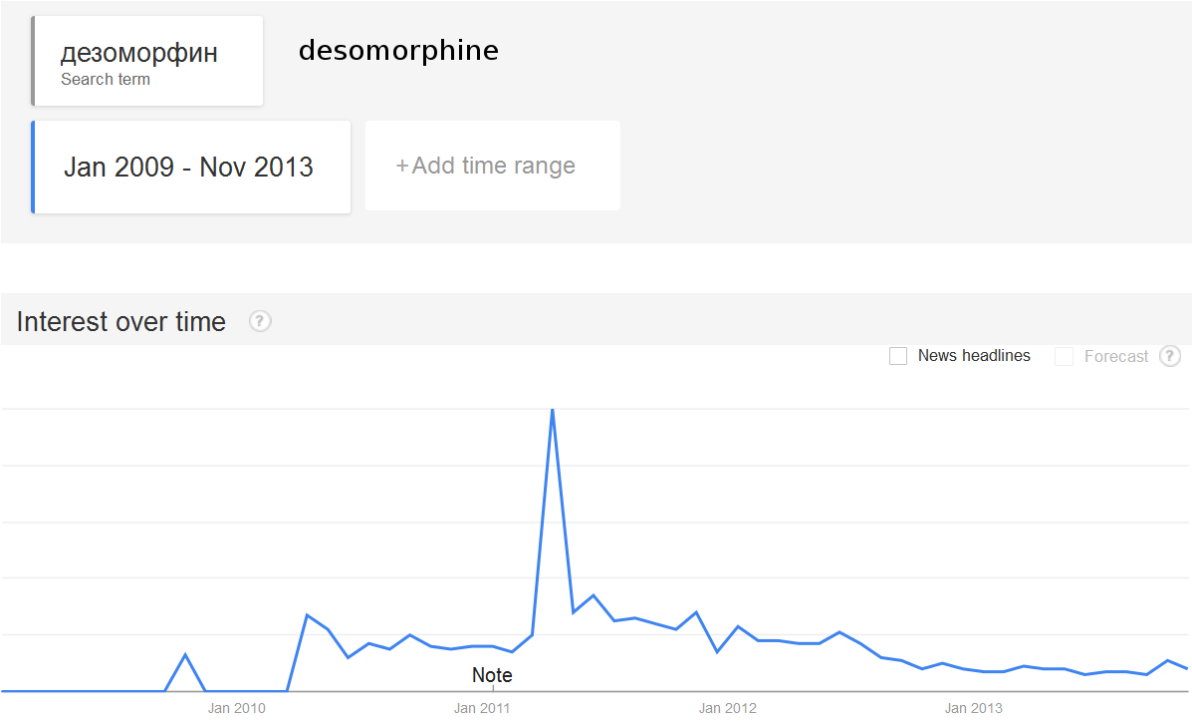
Desomorphine searches Google Trends Russia - 2009-2013.

President Medvedev’s demonstration stimulated increased public interest in krokodil. The FSKN had reported steadily increasing Internet searches in the 12 months before President Medvedev’s speech. However, extensive media coverage and the highest recorded volume of searches for “desomorphine” emerged in the week following the President’s speech (see Figure 1). Further, searches remained consistently elevated after April 2011 until the Russian CCM restrictions in June 2012. Medvedev’s speech may thus have contributed to the Russian government’s unintentional amplification of popular interest in krokodil production and use [47-49]. Both before and after President Medvedev’s speech, Russian media consistently reported the harms of krokodil alongside the low price and ease of access to ingredients, and ease of drug synthesis in domestic laboratories.

Political and public concern over illicit drug use preceded President Medvedev’s speech. Public opinion polls since 2005 consistently rated illicit drug use as one of the most serious social problems in Russia [50]. Between 2010 and 2012, the Russian government progressively tightened restrictions on all illicit drug information available online. For example, in 2011, a website operated by the nongovernmental organization, Andrey Rylkov Foundation, was suspended for publishing public health information about opioid substitution therapy and harm reduction [51]. Russian government policy towards illicit drug use and drug users is regarded as punitive. Moreover, in the post-Soviet period, drug use came to represent an existential threat, implicated in national spiritual and demographic decay (eg, [52]). The intensity of public concern and political action directed at illicit drug use in Russia has been described as a moral panic [53]. Moral panics are a sociological concept describing the disproportionate public response to issues represented in media [54,55]. Over two decades, intense social and political pressures led to the demonization of drug users in Russia. After 2009, krokodil increasingly served as a focus for illicit drug-related concerns. However, President Medvedev’s April 2011 speech may have triggered a moral panic surrounding krokodil, amplified public interest, and stimulated Internet search behavior.

### Public Curiosity Stimulated by Media Reporting on Illicit Drugs

Exposure to stories about illicit drugs in traditional and online media has been found to increase public curiosity and the use of illicit drugs [56,57]. Dasgupta et al found mortality from prescription opiates increased significantly in the months following media reporting [58]. In addition to the news effects, online media has increased information about manufacture, sales, and use of novel illicit drugs, and undermined international drug control efforts [59-61]. Further, researchers have noted the cohort most likely to experiment with new drugs is also most likely to use the Internet [62]. The Internet is now regarded as the main medium from spreading information about novel illicit drugs [63-65]. Notably, Forsythe directly linked news reporting to increased rates of Internet search to increased use of the novel illicit drug mephedrone in the United Kingdom during 2010 [66].

We conducted this exploratory infodemiology study in order to better understand if the relative scale and spatial distribution of search behavior was consistent with an interest in the production and use of krokodil in Russia before and after the imposition of federal restrictions on CCM sales in 2012. In conducting this study, we examined “the science of distribution and determinants of information...(on) the Internet (and) a population, with the ultimate aim to inform public health and public policy” [67].

## Methods

### Objectives

This study had two main objectives: (1) to determine if Internet search patterns could detect regularities in behavioral responses to Russian CCM policy at population level, and (2) if complementary data sources could explain the regularities we observed.

### Search Patterns and Illicit or Stigmatized Behaviors

Each Internet search is a behavioral measure of an issue’s importance to an individual [68]. If individuals are concerned or interested in an issue, they are more likely to search for information related to that issue. The relative importance of an issue can thus be inferred from the volume of search queries for a specific term or terms representing that issue. The use of aggregated Internet search patterns may be considered a method of unobserved, real time behavioral field research at population scale [69]. Researchers have used Internet search patterns to investigate illegal or stigmatized behaviors. For example, researchers analyzed search patterns to monitor US cigarette tax avoidance [70] and the use of racist terms when searching for information about Barak Obama prior to the 2012 US presidential elections [71]. Other investigators have studied global abortion patterns, finding higher search rates in those geographic regions where abortions were illegal or restricted [72]. In each of these instances, the authors suggested Internet search provided insights into behavior at a population level, beyond that available to traditional survey research.

### Google and Yandex Search in Russia

Most Internet search pattern studies have used Google Trends as the data source. Google Trends has been deployed in studies of influenza [73,74], dengue [75], and HIV checks [66,76]. Globally, Google provided 84% of global Internet search queries in May 2011 [77]. In Russia, Google’s market share in Russia was only 25% in 2011 [78], whereas Yandex had a 60% share. The proportion of the Russian population using the Internet grew from 43% in 2010 to 55% in 2012 [79]. Further, there was near universal Internet use among the age groups most likely to use illicit drugs [80]. Among both males and females aged under 35 years and living in major Russian urban centers, more than 90% were regular Internet users during 2012.

### Case Study: Why Is Search-Based Krokodil Surveillance Important in Russia?

Illicit drug use data are generally regarded as difficult to obtain. Drug use estimates are imperfect even in high-income countries with adequate resources [81]. Researchers have generally divided illicit drug use estimation methods into direct and indirect approaches. Direct methods involve surveying household members about patterns of drug use. However, this method is expensive and may not produce truthful responses, particularly in countries such as Russia, where illicit drug use carries severe criminal penalties and stigma [82]. Further, household surveys may fail to reach drug-using populations such as prisoners and the homeless. Indirect methods aim to estimate the size of drug-using populations through comparing data sources. Examples of indirect methods include the multiplier method, based on estimates of the proportion of drug users receiving treatment each year [83]. Additional methods include capture-recapture and back-projection [84], using data sources such as arrest, overdose, and needle exchange data [85,86]. Drug use researchers suggest the main advantage of indirect methods is their lower cost and greater accuracy, as several measures from different data sources are generally combined to produce a single aggregated measure.

In addition, we identified two references to national drug agencies using novel methods for estimating illicit drug use prevalence. The UK government made use of Google Trends when considering restrictions on the novel substance mephedrone in 2010 [66]. Similarly, the Russian FSKN used Internet searches to develop a case for restricting CCM sales in Russia in 2011 [46]. These two instances suggest online search pattern data have been used by national governments in shaping national drug policies. Through conducting this study, we aimed to develop search patterns as an additional method to complement indirect estimates of illicit drug-using populations in Russian-speaking countries.

### RQ1: Were There Regularities in the Internet Search Patterns for “Desomorphine” Across Russia in 2011-2013?

To answer this question, we examined search patterns using Yandex and Google Internet search engines. First, we determined the most appropriate search term to represent the informal term “krokodil” in Internet searches. We initially selected two search terms that we believed reflected the majority of searches for the concept central to this study [87]. These two search terms we selected were “desomorphine” (дезоморфин in Russian) and “Krokodil” (крокодил). We referred to the Google Trends related terms (Top Searches) feature to ensure each term referred to the subject of this study. Google related terms provide information on the relative importance of searches related to the specific search term entered into Google Trends. In the case of “desomorphine”, all terms were related to the drug desomorphine, whereas the term “krokodil” revealed primarily unrelated terms (see Table 3). The term “krokodil” also means “crocodile” in Russian, leading to considerable ambiguity in search results. For example, the most popular term associated with “krokodil” (crocodile) referred to a 1960s Soviet-era children’s animated character “Gena the Crocodile”. Conversely, there is anecdotal evidence to suggest that some Russian PWID may not immediately associate the term krokodil with desomorphine [15]. Overall, based on information available from Google Trends and Russian respondents, we anticipated that the search term “desomorphine” would be more likely to reflect search patterns consistent with an interest in the production and use of krokodil.

**Table 3:** Google Trends related terms for desomorphine, Russian Federation.

Search terms		Russian	Value
**Desomorphine-related**			
	Desomorphine how to prepare	дезоморфин как приготовить	100
	Prepare desomorphine	приготовить дезоморфин	100
	Krokodil desomorphine	крокодил дезоморфин	80
	Krokodil	крокодил	80
	Drug desomorphine	наркотик дезоморфин	60
	Desomorphine recipe	дезоморфин рецепт	50
	Krokodil drug	крокодил наркотик	30
**Krokodil-related**			
	Gena the crocodile (Children’s animation)	крокодил гена	100
	Crocodile game (Children’s game)	игра крокодил	50
	Krokodil drug	крокодил наркотик	50
	Crocodile/krokodil online	крокодил онлайн	45
	Crocodile Dundee (Australian film)	крокодил данди	35
	Dundee (Australian film)	данди	35
	Cheburashka (Children’s animation)	чебурашка	30

Publicly available Google Trends data for Russia has several limitations. First, Google did not provide complete results, returning only oblasts with the highest search volume. Google data for the term desomorphine was available for only 8 of Russia’s 83 oblasts and 3 cities during the date range 2011-2013. Second, Google did not provide raw search data. This made direct comparisons between oblasts using Google data impossible. We thus used WordStat as the primary data source. Yandex made publicly available a complete raw search dataset for all Russian regions and oblasts for 6 months before and after the implementation of federal CCM restrictions in June 2012. We used Google Trends as a secondary source of aggregated search results for validation purposes.

Second, we obtained desomorphine search data for each Russia oblast from September 1, 2011, to August 31, 2013. Yandex provides 2 years of publicly available monthly search pattern data at any time. Additionally, we had 6 months previously downloaded monthly search pattern data for the term desomorphine for each Russian oblast, from February to August 2011.

Third, we converted raw search figures for the term desomorphine to population prevalences. This allowed direct comparison across regions and oblasts. We used 2010 federal Russian census data [88] for our population prevalence calculation. We then multiplied each result by 100,000 to increase ease of comprehension, and to provide a population prevalence measure.

Fourth, we analyzed search patterns before and after federal restrictions on CCM sales in June 2012. We obtained the mean search volume for 6 months before the restrictions, as well as 6 and 12 months after (ie, to August 31, 2013). We excluded June 2012 data, as we anticipated atypical search patterns in the immediate post-restriction period. Overall, we segmented the available data to examine the effects of a federal policy change on the relative scale and geographic patterns of krokodil search across the Russia.

Fifth, we obtained all available Google data for the term desomorphine from September 2011 to August 2012. Google search data for the term desomorphine was available for 8 of 83 oblasts only (see Table 4). We did not consider this sample adequate to conduct correlations. Similar limitations with regional Russian Google search results have been reported in earlier studies [69,89].

**Table 4:** All available Google Trends results in Russia from September 2011 to September 2013.

Region		Search volume
**Oblast**		
	Chelyabinsk	100
	Novosibirsk	88
	Sverdlovsk	86
	Samara	85
	Rostov	83
	Saint Petersburg city	71
	Moscow city	68
	Krasnodar	67
**City**		
	Yekaterinburg	100
	Nizhny Novgorod	98
	Chelyabinsk	89
	Samara	87
	Novosibirsk	87
	Rostov-on-Don	85
	Saint Petersburg	81
	Moscow	77
	Kazan	68
	Krasnodar	61

### RQ2: Can Complementary Data Sources Explain the Observed Regularities for “Desomorphine” Across Russia in 2011-2013?

To answer this question, we initially reviewed the approaches used to validate search pattern data and drug population data. Search pattern studies have generally validated against an offline measure. For example, the initial search pattern studies established correlations between search patterns and epidemiological surveillance data for influenza [70,90]. Other studies focusing on issue salience, established a correlation between search patterns, traditional media, and opinion polling [91]. Each of these search studies revealed regular patterns of behavior corresponding to a valid offline measure. However, in this case, there was no analogous source of illicit drug use data available. We therefore combined several complementary data sources with a view to providing a plausible explanation for the observed regularities in Internet search patterns.

First, we obtained first court appearance data available for krokodil-related criminal charges for the 77 of 83 Russian oblast data available from the Rospravosudie website. The site is a publicly available, non-government Russian criminal justice research project displaying criminal court case data across all Russian oblasts [16]. One part of the project is dedicated to court appearance data for popular illicit recreational drugs. In addition to krokodil, Rospravosudie provides arrest data on 24 illicit drugs, including krokodil marijuana, amphetamines, JWH (“spice”), and heroin. The available krokodil data is a single per-oblast figure covering the date range 2010 to 2012. The Rospravosudie website publishers note several limitations. First, the arrest data are based on sentencing documents. Only 50% of complete sentencing data are published, and only 50% of cases appear in courts. Second, the database allows comparison of the relative popularity of various illicit drugs. It is not possible to describe the absolute prevalence of illicit drug use based on these data. Third, there is an assumption that the detection rates for illicit drug crimes were on average the same nationally. Fourth, only the first court appearances for illicit drug cases are recorded. Subsequent appeals and cassations for illicit drugs are excluded. In summary, the site authors suggest that despite these limitations, the data reflect the differences in access to illicit drugs across Russian regions.

To analyze krokodil-related court data, we first obtained arrest rates for krokodil for 2010-2012 as a single figure for each Russian oblast. We then converted the arrest rates for each oblast to a per 100,000 population measure. This allowed us to investigate the relationship between court appearances and krokodil searches. We used the mean searches for “desomorphine” per 100,000 population from November 2011 to May 2012 to represent pre-CCM restriction searches. We then conducted Spearman correlation between arrest rates and searches per 100,000 population for “desomorphine” for the 77 regions for which court data were available.

Second, we used Google Trends visual data to provide indicative national search results for popular CCMs and “desomorphine” from January 2009 to January 2013. We identified several popular CCM available in Russia prior to the June 2012 ban [92]. The four CCM we analyzed with Google Trends were kaffetin, solpadeine, pentalgin, and codelac. Historical Yandex data were not available for this complete date range.

Third, we used Google Trends related searches to analyze several popular CCM available in Russia prior to the June 2012 restrictions. Through analyzing these related searches, we sought to obtain additional information on the characteristics of public interest in CCM and the term desomorphine before and after federal restrictions. Historical Yandex data were not available for this complete date range.

Fourth, we used Yandex keyword feature nationally to confirm that searches for the term desomorphine were associated with illicit drug use. Yandex provides a keyword function that lists word combinations associated with a specified search term. Keywords are analogous to the Google related terms (Top Searches) feature [93]. We identified 85 Yandex keyword combinations incorporating the term desomorphine. These combinations included “desomorphine prepare” and “desomorphine recipe” (see Multimedia Appendix 1).Yandex keywords are available nationally, for each Russian region and 83 oblasts, and many smaller intra-oblast cities. However, the date range is limited to the preceding 30 days only. As the search patterns from month to month are likely to be volatile within smaller geographic units, we obtained and analyzed the results for Yandex national keywords only. In order to identify the main themes present in keyword results, we hand coded the 85 keyword combinations from the national level Yandex keyword feature in the latest available date range, November 2013. Two Russian-speaking researchers then coded each keyword combination into one of three primary themes.

## Results

### RQ1: Were There Regularities in the Internet Search Patterns for “Desomorphine” Across Russia in 2011-13?

In the 6 months before the CCM restrictions in June 2012, 21 of Russia’s 89 oblasts had Internet search rates higher than the national average (mean) of 16.67 per 100,000 (see Multimedia Appendix 2). In the 6 months immediately after restrictions, national average search rates dropped to 9.65 per 100,000. Further, the number of oblasts with a higher than average search rate dropped from 30 to 16. In the 6-month date range from March to August 2013, search rates dropped further to 8.75 per 100,000, with 11 oblasts recording higher than average search rates. However, there were a number of oblasts where searches for “desomorphine” persisted after the federal restriction on CCM sales. These included Sverdlovsk oblast (146.898 before CCM restrictions vs 81.098 post restrictions), Moscow city (31.245 vs 20.586), and Vologda oblast (34.061 vs 17.998). See Table 5. A further detailed analysis of subnational search pattern results appears in narrative form in Multimedia Appendix 3.

**Table 5:** Yandex search patterns for “desomorphine” in selected Russian subregional cities.

Cities		Pre-ban, 6 months, Dec 2011-June 2012	Post-ban, 6 months, July-Dec 2012	Post-ban, 6 months, Feb-Sept 2013	% change post-ban, 6 months	% change, Feb-Sept 2013
**Vologda Oblast**						
	Vologda city	80.916	28.389	33.857	64.915	58.157
	Cherepovets	50.324	28.337	25.829	43.690	48.674
**Sverdlovsk Oblast**						
	Yekaterinburg	16.200	10.372	10.261	35.976	36.662
	Kamensk-uralskiy	20.226	13.262	6.583	34.434	67.453
	Pervouralsk	13.116	6.290	6.157	52.041	53.061
**Rostov Oblast**						
	Rostov-na-donu	56.460	43.409	42.353	23.117	24.986
	Kamensk-Shakhtinsky	17.850	13.002	5.950	27.160	66.667
	Shakhty	2.778	0.764	1.875	72.500	32.500
	Volgodonsk	26.633	11.219	8.780	57.875	67.033
	Taganrog	13.324	6.080	8.861	54.369	33.495
	Novocherkassk	6.420	4.247	3.852	33.846	40.000
**Samara Oblast**						
	Samara city	25.329	16.442	24.227	35.085	4.350
	Togliatti	30.131	18.736	15.147	37.817	49.731
**Krasnodar Oblast**						
	Sochi	16.068	9.320	8.495	41.994	47.130
	Novorossiysk	11.986	11.159	3.582	6.897	70.115
	Krasnodar city	32.774	20.917	14.586	36.177	55.495

### RQ2: Can Complementary Data Sources Explain the Observed Regularities for “Desomorphine” Across Russia in 2011-13?

To answer this question, we used several complementary sources of krokodil-related data. We found a Spearman correlation of .506 (*P*≤.001) between searches for the term “desomorphine” and first court appearance data for krokodil related charges for all Russian oblasts. That is a moderately strong positive correlation (see Table 6).

**Table 6:** Correlation between searches for the term desomorphine and court appearances.

No. of subregions	Data source	Date range	Spearman correlation
83 (77 correlated)	“desomorphine” searches, Yandex WordStat	Dec 2011-May 12	—
77	desomorphine court appearances	2010-2012	.506 (*P*≤.001)

Second, we examined national Google Trends results for four CCMs and “desomorphine”. Overall, search volumes for both CCM decreased in the 6 months before the June 2012 federal restrictions, as did searches for the term “desomorphine”. Public interest in CCM and the term desomorphine was roughly similar in the 6 months before the implementation of restrictions. The exception was an increase in search for the CCM pentalgin immediately before the June 2012 restrictions (see Table 7). We attribute this marked increase in interest due to public concern over access to CCM for therapeutic analgesic purposes.

Third, we examined Google Trends related terms for CCMs and desomorphine. We found related terms for CCMs consistent with therapeutic and analgesic uses (see Table 7). By contrast, related terms for desomorphine were consistent with an interest in the production and use of krokodil. The Google related terms data did not record all search results. We attribute this to the Google “threshold effect” described in earlier analysis of drug policy [69]. That is, below an unspecified threshold value, Google records a nil value.

**Table 7:** Google related search terms for “desomorphine” from 2009-2013.

Date range	Pentalgin (пенталгин)	Value	Codelac (коделак)	Value	Desomorphine (дезоморфин)	Value
2009-2013	Pentalgin N	100	Codelac broncho	100	Desomorphine how to prepare	100
	Pentalgin instructions	65	Codelac phyto	75	Desomorphine krokodil	75
	Pentalgin composition	50	Codelac instructions	75	Krokodil	70
	Pentalgin price	45	Codelac price	65		
	Nurofen	40	Codelac syrup	60		
Dec 2011-May 2012	Pentalgin N	100	Codelac phyto	100	How to prepare desomorphine	100
	Pentalgin instructions	85	Codelac instructions	100	Krokodil	55
July 2012-Aug 2013	Insufficient search volume	Nil	Insufficient search volume	Nil	Insufficient search volume	Nil

Fourth, we used the Yandex keyword feature to analyze the word combinations used with the search term desomorphine. We found combinations associated with krokodil preparation and use accounted for 46.613% of searches, images, and general information for 24.175%, and ambiguous terms for 29.212% (see Multimedia Appendix 1 and Table 8). We used Cohen’s Kappa to assess intercoder reliability on all 85 search combinations across three categories using two coders (kappa=.772).

The preparation and use category included all terms associated with drug preparation and use. Images and entertainment included visual material and terms unlikely to be associated with drug use and preparation (eg, “YouTube desomorphine”, “junkies desomorphine”). In summary, we found the combination of search patterns with complementary methods useful for identifying behaviors consistent with an interest in the production and use of krokodil.

**Table 8:** Main themes identified in WordStat keyword combined word searches for “desomorphine” (excluding non-combined word searches for the single term “desomorphine”.

Code		n (N=6338)	Percentage
1	Preparation & Use	2952	46.613
2	Images & information	1531	24.175
3	Ambiguous	1850	29.212

## Discussion

### Principal Findings

We found federal CCM restrictions in June 2012 coincided with changes in the relative scale and spatial patterns of Internet search behaviors consistent with an interest in the production and use of krokodil. These changes in Internet search appeared consistent with behaviors that may be anticipated in the production and use of krokodil in response to changed access to CCM.

We observed marked reductions in searches for the term desomorphine following CCM sales restrictions in June 2012. By comparison with the 6 months preceding federal restrictions, searches dropped by 42.095% nationally (see Multimedia Appendix 2). However, the patterns of decreased search for “desomorphine” varied considerably. This suggests that CCM restrictions changed but did not extinguish behaviors consistent with an interest in the production and use of krokodil.

Third, we found the Google data available were inadequate for statistical analysis. Insufficient Google Trends data were available to conduct statistical analysis to identify oblasts where krokodil use may be prevalent. Google Trends data were available for only 8 of 83 regions (see Table 4).

We identified several complementary data sources that provided a plausible explanation for the observed regularities in Internet search data. First, we found a moderately strong positive correlation (Spearman correlation=.506) between the geographic distribution of court appearances for krokodil-related charges, and Internet searches for the term desomorphine. This result should be treated with some caution. Court appearance data were available for 78 of 83 statistical regions. This may have affected the strength of correlations. More significantly, international researchers generally regard Russian policing as predatory and beyond the rule of law [94,95]. A 2010 study of PWID found reports of evidence planted by police, extortion money, arbitrary arrests, and violence to be widespread. Further, Russian court processes are regarded by researchers and Russian public opinion as likely to produce outcomes favoring police and prosecutors [96-98]. Both policing and judicial practices may be expected to distort court appearance data. It is possible that in the absence of these law enforcement practices the strength of correlation may differ. Conversely, the uncertainty surrounding Russian law enforcement data is consistent with descriptions of other data sources used for indirect drug population estimation by international researchers.

**Figure 2:**
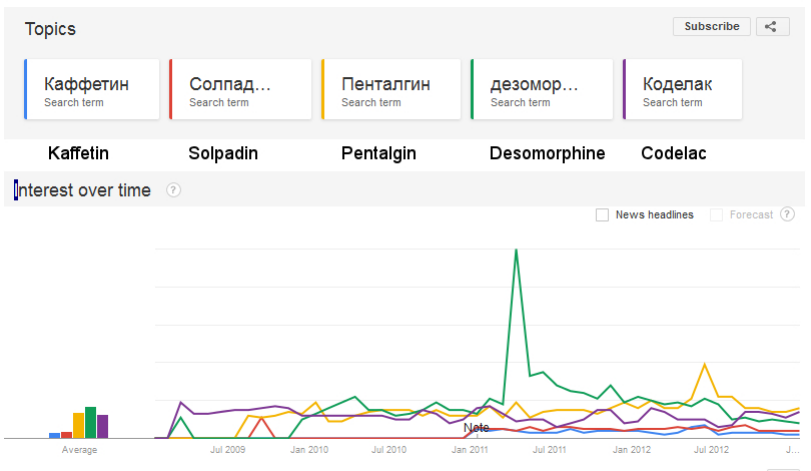
Google Trends results for CCM and desomorphine search 2009 - 2013.

Second, the available Google Trends data suggested public interest in CCM and the term desomorphine was roughly similar in the 6 months prior to federal restrictions. However, the searches for CCM and desomorphine-related terms were not identical. The interest in CCM and in desomorphine manifested as different national level search patterns over the date range. While we had insufficient Google data to conduct correlations, this difference is evident on visual inspection (see Figure 2). Similarly, Google Trends related terms results suggest different themes for “desomorphine” and CCM (see Table 7). Whereas searches for the term desomorphine were associated with illicit drug use themes, the CCM search themes were primarily associated with therapeutic use of drugs. Finally, there is anecdotal evidence from Russian informants that CCM was also widely consumed orally rather than injected prior to the introduction of federal restrictions (P. Meylakhs, Personal Communication, 2014). The restrictions on CCM sales thus also affected oral use of CCM as a recreational drug.

Yandex keyword analysis revealed a consistent pattern of behavior that was consistent with an interest in the production and use of krokodil. Yandex keyword data also revealed a strong popular interest in visual images of desomorphine use (see Table 8). Overall, 23.59% of searches were coded as pertaining to the images and general information theme. These graphic images were actively employed in Russian government campaigns aimed at reducing krokodil use. However, the wide distribution of images may also have created a popular demand for viewing necrotic injuries as voyeurism or entertainment. While the available Yandex keyword data were outside of the date range of the study, they reveal persistent interest in behavior consistent with an interest in the production and use of krokodil in Russia.

In summary, we used complementary data sources in order to investigate behaviors consistent with an interest in the production and use of krokodil. Our analysis suggests that these combined complementary sources, including online news sources, provided a useful addition to the conventional approaches used to analyze krokodil use in Russia. Further, our analysis also suggests it is plausible that Yandex search behavior served as a proxy for krokodil production and use in the date range 2011-2012.

### Further Research and Limitations

Our research suggests that further research into the use of search patterns for investigating illicit drug use prevalence is warranted. First, search patterns offer researchers and non-government groups an additional source of indirect data with which to track the prevalence of traditional and emerging synthetic drugs at low cost and in near real time. We identified two references to national drug agencies in United Kingdom and Russia using Internet search methods to research patterns of illicit drug use [46,66]. Search pattern methods offer non-government groups similar capabilities. Further, the methods described in this paper are directly applicable to the study of other illicit drugs and in Russian-speaking countries where Yandex is widely used alongside Google.

Second, the krokodil case represents an example of a broader class of illicit drug policy events. International and Russian researchers have partially attributed increased use of krokodil to decreased heroin supply after 2009. Similarly, in 2012, government policy blocked easy access to CCM. In each case, existing networks of PWID were disrupted, and patterns of illicit drug use rapidly changed [18]. Combining Internet search and offline qualitative methods would extend understanding of rapid shifts in illicit drug markets and potentially improve public health responses to emerging synthetic drugs. This qualitative research may include existing drug use prevalence, strength of the heroin market, and Internet access among PWID in Russia. In particular, this research would assist researchers with discerning rapid shifts in drug use patterns in response to policy changes and other external shocks to existing illicit drug markets. The relationship of searches for "desomorphine" to other illicit drugs appears in Multimedia Appendices 4 and 5.

Third, media censorship is increasing in contemporary Russia. However, our analysis of online information relied on measures of unobserved population level demand for online information only. By contrast, censorship may be expected to influence the supply of illicit drug-related information. Russian government actions restricting the supply of illicit drug information are well documented in international literature (eg, [51,99]). Future research should investigate the relationship between searches for information and the censored supply of information.

Finally, search methods do not estimate actual drug user population size. However, our research suggests search methods can complement existing drug-using population estimation methods. For example, the Yandex keywords feature potentially provides a novel data source with which to track monthly shifts in keywords for illicit drug–related terms. Keywords measures provide a low-cost method for identifying spatial shifts in the relative scale of public interest in terms that are consistent with an interest in the production and use of novel and emerging illicit drugs in an increasingly complex environment where the opportunities for conventional field work and surveys in Russia for international researchers are decreasing.

### Conclusions

Illicit drug use data are generally regarded as difficult to obtain through traditional survey methods. We used complementary methods to explain observed regularities in patterns of Internet search behavior before and after the imposition of Russian federal restrictions on CCM sales in 2012. Our analysis suggests it is plausible that Yandex search behavior served as a proxy for patterns of krokodil production and use during the date range we investigated. More generally, this study demonstrates the application of novel methods recently used by policy makers to both monitor illicit drug use and influence drug policy decision making.

## Multimedia Appendix 1

Yandex Wordstat Keywords: combinations of words searched for with "desomorphine", November 2013.

## Multimedia Appendix 2

Per-capita mean searches for "desomorphine" in all Russian federal regions & subregions (**= region or subregion above national mean).

## Multimedia Appendix 3

Analysis of regional Internet search patterns.

## Multimedia Appendix 4

Illicit drug search popularity of related terms in Google Trends 2009-2014.

## Multimedia Appendix 5

Google searches for popular illicit drugs in the Russian Federation 2009-2014.

## Abbreviations

CCM: codeine-containing medication
HIV: human immunodeficiency virus
PWID: people who inject drugs
FSKN: Russian Federal Drug Control Service
