# Addition to Acknowledgments: Internet Search and Krokodil in the Russian Federation: An Infoveillance Study

**DOI:** 10.2196/jmir.5487

**Published:** 2016-01-19

**Authors:** Andrey Zheluk, Casey Quinn, Peter Meylakhs

**Affiliations:** ^1^ Menzies Centre for Health Policy University of Sydney Sydney Australia; ^2^ PRMA Consulting Ltd Fleet, Hampshire United Kingdom; ^3^ Laboratory for Internet Studies National Research University Higher School of Economics St Petersburg Russian Federation

The authors of the published article “Internet Search and Krokodil in the Russian Federation: An Infoveillance Study” (J Med Internet Res 2014;16[9]:e212) wish to add additional information to the “Acknowledgments” section, so it now reads as follows:

The work of Peter Meylakhs was funded by the Basic Research Program of the National Research University Higher School of Economics, Russia. The authors would also like to thank Ms Svetlana Chernova in Ukraine for her assistance with data collection and coding, Associate Professor James Gillespie at the Menzies Centre for Health Policy, University of Sydney for his advice and support, and Ms Anya Sarang from the Andrey Rylkov Foundation in Moscow for facilitating contacts within Russia.

The first sentence was unintentionally omitted in the original submission. The publication on the JMIR site was amended accordingly, and a new version was submitted to PubMed Central. 

