# Supplementary material for: Internet Search and Krokodil in the Russian Federation: An Infoveillance Study
Source: J Med Internet Res. 2014 Sep 18;16(9):e212. doi: 10.2196/jmir.3203 (PMC4180331; doi:10.2196/jmir.3203)
Supplement: Supplementary file 1 [file jmir_v16i9e212_app1.pdf]

Codes 1=Preparation & Use,   2 = Images & information, 3= Ambiguous

Russian term	English term	Searches	Code
дезоморфин состав	Components desomorphine	334	1
приготовление дезоморфина	Preparation desomorphine	330	1
дезоморфин изготовление	Desomorphine preparation	214	1
дезоморфин рецепт	Desomorphine recipe	260	1
употребление дезоморфина	Consumption desomorphine	272	1
как приготовить дезоморфин	How to prepare desomorphine	160	1
дезоморфин способ	Desomorphine method	229	1
как варить дезоморфин	How to cook desomorphine	166	1
дезоморфин +как варить	Desomorphine how to cook	134	1
состав дезоморфина	Components desomorphine	124	1
дезоморфин +как сделать	Desomorphine how to make	121	1
способ приготовления дезоморфин	Method of preparing desomorphine	115	1
как изготовить дезоморфин	How to prepare desomorphine	111	1
дезоморфин способ изготовления	Desomorphine method preparation	101	1
как сварить дезоморфин	How to cook up desomorphine	94	1
как сделать дезоморфин	How to make desomorphine	92	1
синтез дезоморфина	Synthesis desomorphine	39	1
дезоморфин способ приготовления	Desomorphine method of preparation	80	
дезоморфин форум	Desomorphine forum	77	1
коаксил дезоморфин	Coaxil desmorphine	63	1
как готовить дезоморфин	How to prepare desomorphine	52	1
получение дезоморфина	Getting desomorphine	56	1
дезоморфин +в домашних условиях	Desomorphine in domestic conditions	54	1
из чего делают дезоморфин	What is desomorphine made from	51	1
действие дезоморфина	Desomorphine action	25	1
дезоморфин запах	Desomorphine smell	45	1
дезоморфин купить	Desomorphine buy	43	1
рецепт приготовления дезоморфина	Recipe for preparing desomorphine	39	1
дезоморфин лечение	Desomorphine treatment	38	1
сухой остаток дезоморфин	Drug residue desomorphine	38	1
сколько держится дезоморфин +в моче	How long is desomorphine detectable in urine	33	1
из чего делают дезоморфин	What is desomorphine made from	31	1
рецепт изготовления дезоморфина	Recipe for preparing desomorphine	31	1
под дезоморфином	Stoned on desomorphine	26	1
колется дезоморфином	Injecting desomorphine	23	1

дезоморфин эффект	Desomorphine effect	23	1
сколько держится +в крови дезоморфин	How long is desomorphine detectable in blood	10	1
дезоморфин инструкция	Desomorphine instructions	21	1
сбыт дезоморфина	Sale desomophine	23	1
как приготовить дезоморфин видео	How to prepare desomorphine video	8	1
дезоморфин реакция	Desomorphine reaction	18	1
дезоморфин ингредиенты	Desomorphine ingredients	18	1
дезоморфин приход	Desomorphine effects	16	1
варка дезоморфина	Cooking desomorphine	15	1
дезоморфин ощущения	Desomorphine feelings	14	1
признаки употребления дезоморфина	Signs of desomorphine use	15	1
дезоморфин симптомы	Desomorphine symptoms	13	1
дезоморфин формула	Desomorphine formula	11	1
производство дезоморфина	Production of desomorphine	15	1
инструкция +по изготовлению дезоморфина	Instructions for preparing desomorphine	7	1
как избавиться от запаха дезоморфина	How to get rid of the smell of desomorphine	7	1
дезоморфин цена	Desomorphine price	6	1
дезоморфин видео	Desomorphine video	673	2
дезоморфин фото	Desomorphine photo	206	2
наркоманы дезоморфин	Junkies desomorphine	155	2
последствия употребления дезоморфина видео	Consequences of desomorphine use video	117	2
последствия дезоморфина фото	Consequences of desomorphine use photo	69	2
последствия приема дезоморфина	Consequences of taking desomorphine	61	2
фильм дезоморфин	Desomorphine film	61	2
смотреть видео дезоморфин	Watch video desomorphine	25	2
дезоморфин смотреть онлайн	Desomorphine watch online	19	2
видео наркоманы дезоморфин	Video junkies desomorphine	27	2
наркоманы употребляющие дезоморфин	Junkies using desomorphine	26	2
смотреть +про дезоморфин	Watch about desomorphine	20	2
дезоморфин наркотик видео	Desomorphine drug video	18	2
ютуб дезоморфин	Youtube desomorphine	23	2
дезоморфин видео онлайн	Desomorphine video online	10	2
крокодил дезоморфин видео	Krokodil desomorphine video	13	2
дезоморфин документальный фильм	Desomorphine documentary film	11	2
крокодил дезоморфин	Krokodil desomorphine	232	3
последствия дезоморфина	Consequences desomorphine	748	3
наркотик дезоморфин	Drug desomorphine	199	3

дезоморфин крокодил	Desomorphine krokodil	327	3
последствия дезоморфина видео	Consequences desomorphine video	186	3
последствия употребления дезоморфина	Consequences of consuming desomorphine	209	3
человек дезоморфин	Person desomorphine	92	3
скачать дезоморфин	Download desomorphine	54	3
дезоморфин 2013	Desomorphine 2013	52	3
кодеин дезоморфин	Codeine desomorphine	51	3
онлайн дезоморфин	Online desomorphine	47	3
дезоморфин википедия	Desomorphine wikipedia	43	3
дезоморфин крокодил наркотик	Desomorphine krokodil drug	43	3
остеомиелит дезоморфин	Osteomyelitis desomorphine	33	3
дезоморфин остеонекроз	Desomorphine ostreonecrosis	29	3
жертвы дезоморфина	Victims of desomorphine	30	3
смерть +от дезоморфина	Death from desomorphine	24	3
дезоморфин лурк	Desomorphine lurk	20	3
последствия после дезоморфина	Consequences after desomorphine	19	3
как выглядит дезоморфин	What does desomorphine look like	11	3
последствия применения дезоморфина	Consequences using desomorphine	11	3
наркотик дезоморфин крокодил	Drug desomorphine krokodil	8	3
кто придумал дезоморфин	Who thought up desomorphine	8	3
	Total word combination searches	6333	
	Single word searches “desomorphine”	5701	-
	Total all searches November 2013	12034	-
