# Supplementary material for: Internet Search and Krokodil in the Russian Federation: An Infoveillance Study
Source: J Med Internet Res. 2014 Sep 18;16(9):e212. doi: 10.2196/jmir.3203 (PMC4180331; doi:10.2196/jmir.3203)
Supplement: Supplementary file 2 [file jmir_v16i9e212_app2.pdf]

	Pre -Ban<br>6 - 0 months<br>Dec 11-May 12	Post - ban<br>1- 6 months<br>Mean<br>July 12 -Dec 12	Post - ban<br>1- 6 months<br>% change<br>July 12 -Dec 12<br>(relative to pre-ban)	Post-ban<br>8 -13 months<br>Mean<br>March-August 2013	Post-ban<br>8 -13 months<br>cumulative<br>% change<br>March-August 2013<br>(relative to pre-ban)
**NATIONAL**	**16.667**	**9.651**	**- 42.095**	**8.747**	**-47.519**
Central	14.389	7.930	-55.111	6.544	-54.520
Belgorod	10.161	5.590	-44.989	3.965	-60.981
Bryansk	**20.704	**10.656	-48.529	7.148	-65.473
Vladimir	12.230	6.495	-46.896	5.140	-57.975
Voronezh	9.953	7.207	-27.586	5.641	-43.319
Ivanovo	11.460	8.068	-29.599	8.337	-27.248
Kaluga	9.518	5.628	-40.870	4.320	-54.609
Kostroma	**18.638	6.290	-66.252	7.051	-62.167
Kursk	**22.060	8.420	-61.834	7.839	-64.464
Lipetsk	13.416	9.292	-30.736	5.469	-59.232
Moscow Region	4.482	3.947	-11.949	2.269	-49.389
Orel	12.737	**10.061	-21.008	5.159	-59.496
Ryazan	**22.851	**12.211	-46.565	**12.080	-47.137
Smolensk	9.766	4.415	-54.799	4.721	-51.658
Tambov	12.896	5.699	55.808	3.660	-71.617
Tver	8.345	4.534	-45.672	3.786	-54.627
Tula	10.898	5.427	-50.199	4.084	-62.525
Yaroslavl	**17.645	8.206	-53.492	6.253	-64.562
MoscowCity	**31.245	**20.586	-34.114	**20.874	-33.192
North Western Federal Region	16.293	9.134	-43.939	6.552	-59.787
Karelia	16.398	6.684	-59.236	3.421	-79.140
Komi	**26.622	**14.968	-43.777	**11.447	-57.001
Archangelsk	16.295	8.927	-45.216	7.934	-51.312
Nenets Autonomous Region	5.476	4.302	-21.429	2.738	-50.000
Vologda	**34.061	**17.998	-47.160	**15.152	-55.516
Kaliningrad	8.642	5.855	-32.252	4.347	-49.696
Leningrad	1.645	1.110	-32.558	1.138	-30.814
Murmansk	**28.948	**13.517	-53.304	**8.778	-69.677
Novgorod	12.132	7.407	-38.950	5.256	-56.674
Pskov	6.825	3.111	-54.412	2.534	-62.868
St. Petersburg City	**22.185	**16.598	-25.184	**9.328	-57.956
Southern Federal Region	8.220	5.007	-39.091	4.501	-45.245
Adygia	2.819	1.842	-34.667	2.142	-24.000
Kalmykia	2.336	1.401	-40.000	2.453	-5.000
Krasnodar	7.402	4.610	-37.717	3.344	-54.815
Astrakhan	9.349	4.338	-53.603	3.713	-60.281
Volgograd	9.895	5.215	-47.300	2.968	-70.007
Rostov	**17.521	**12.636	-27.882	**12.386	-29.312
North Caucuses	3.168	2.278	-28.105	2.272	-28.282

Federal Region					
Dagestan	6.126	3.880	-36.667	3.279	-46.481
Ingushetia	0.115	0.115	0.000	0.420	-266.667
Kabardino-Balkaria	1.882	2.348	-24.742	2.154	-14.433
Karachayevo-Cherkessiya	1.620	1.831	-13.043	2.782	-71.739
Northern Ossetiya	6.713	4.004	40.351	3.934	-41.404
Chechnya	0.964	0.952	1.316	0.964	0.000
Stavropol	4.757	2.815	-40.829	2.372	-50.126
Volga Federal Region	14.789	7.755	-47.563	6.852	-53.669
Bashkortostan	16.562	9.169	-44.637	7.196	-56.552
MariyEl	10.679	7.304	-31.603	4.869	-54.402
Mordovia	7.259	3.427	-52.793	6.387	-12.011
Tatarstan	6.583	3.331	-49.402	2.925	-55.578
Udmutriya	12.484	6.193	-50.396	4.645	-62.797
Chuvashiya	10.667	6.987	-34.504	6.023	-43.538
Perm	**29.468	**13.053	-55.704	**9.705	-67.068
Kirov	8.286	4.659	-43.769	5.125	-38.146
Nizhegorod	**18.633	**11.604	-37.724	9.362	-49.756
Orenburg	**21.561	7.616	-64.677	7.822	-63.720
Penza	13.600	7.383	-45.714	6.326	-53.482
Samara	**16.757	10.668	-36.335	**12.405	-25.967
Saratov	**19.160	8.440	-55.953	6.238	-67.442
Ulyanovsk	15.346	8.736	-43.076	6.897	-55.055
Urals Federal Region	**35.290	**19.335	-45.209	**13.478	-61.807
Kurgan	10.104	7.018	-30.540	4.384	-56.611
Sverdlovsk	**146.898	**81.098	-44.793	**54.304	-63.033
Tyumen	8.669	3.741	-56.846	2.768	-68.072
Khanty-Mansiysk	**20.159	**11.952	-40.710	**10.132	-49.739
Yamalo-Nenetsk	11.632	5.785	-50.265	4.647	-60.053
Chelyabinsk	14.274	6.417	-55.042	4.634	-67.538
Siberian Federal Region	12.428	6.073	-51.134	4.475	-63.995
Altay Republic	2.615	1.268	-51.515	0.713	-72.727
Buryatiya	6.328	2.744	56.640	3.001	-52.575
Tyva	3.060	0.966	-68.421	0.752	-75.439
Khakasiya	14.665	8.067	-44.989	6.723	-54.158
Altay Region	**17.801	8.060	-54.723	5.892	-66.901
Zabaykal	12.586	4.368	-65.296	3.470	-72.430
Krasnoyarsk	**26.319	**12.887	-51.034	7.056	-73.192
Irkutsk	7.879	4.521	-42.620	4.280	-45.677
Kemerovo	8.004	4.582	-42.749	3.871	-51.632
Novosibirsk	**20.871	**12.383	-40.672	8.495	-59.299
Omsk	14.168	6.847	-51.669	4.796	-66.150
Tomsk	14.846	6.186	-58.333	4.651	-68.671
Far East Federal Region	6.663	3.896	-41.538	2.500	-62.475
Sakha (Yakutiya)	6.087	1.866	-69.341	1.953	-67.908
Kamchatka	3.952	3.484	-11.842	1.560	-60.526
Primorsk	9.809	4.451	-54.625	4.006	-59.162

Khabarovsk	12.294	**9.798	-20.303	7.352	-40.202
Amursk	3.836	1.755	-54.255	1.306	-65.957
Magadan	3.829	2.297	-40.000	0.328	-91.429
Sakhalin	14.393	7.027	-51.174	4.358	-69.718
Jewish Autonomous Region	5.116	2.413	-52.830	1.641	-67.925
Chukhotka	0.656	1.969	+200.000	0.000	100.000
