# Supplementary material for: Internet Search and Krokodil in the Russian Federation: An Infoveillance Study
Source: J Med Internet Res. 2014 Sep 18;16(9):e212. doi: 10.2196/jmir.3203 (PMC4180331; doi:10.2196/jmir.3203)
Supplement: Supplementary file 3 [file jmir_v16i9e212_app3.pdf]

**Appendix 3**
**Analysis of regional internet search patterns for behaviour consistent with an interest in the production and use of krokodil**

We conducted additional analysis to demonstrate the complementary use of Google and Yandex data in analysing regional internet search patterns for behaviour consistent with an interest in the production and use of krokodil. We initially noted a discrepancy between Google and Yandex results at the oblast level. Specifically, between 2012-13, Yandex suggested search rates in Krasnodar oblast were lower than the national average. See Figure 6. Google data suggested Krasnodar oblast had among the highest search rates in Russia, whereas Yandex suggested Krasnodar oblast was 7.402 ie below the national average. See Figure 5 and 6 respectively.

We were able to investigate this discrepancy, as Yandex Wordstat provided search localised to the level of provincial cities within oblasts. We identified specific urban centres within Russian oblasts with high volumes of search for the term "desomorphine". For example, the southern Russian Krasnodar oblast recorded lower than national average search rates in the 6 months pre and post the June 2012 CCM ban. See Appendix 2. In the 6 months pre-ban, Krasnodar oblast record 7.402 searches per 100,000 (pre-ban national average 16.667) and 4.610 in the 6 months post-ban (post-ban national average 9.651). By contrast, Krasnodar city recorded 32.774 searches in the 6 months pre-ban, and 20.917 in the 6 months post-ban. Other cities in Krasnodar region such as Sochi (Sochi – 16.068 pre-ban, 9.320 post-ban) and Novorossiysk (11.986 pre-ban, 11.159 post-ban) recorded considerably lower search rates over the date range. In addition to Krasnodar region, we identified similar effects in Vologda oblast (Vologda and Cherepovets cities), and Samara oblast (Samara and Togliatti cities) See Figure 6. The raw data available through Yandex search results are of particular value in identifying the relative scale and spatial patterns of behaviours consistent with an interest in the production and use of krokodil at different administrative tiers.

Our research suggests that search for illicit drug use patterns varies considerably between oblasts, and between urban centres within a oblast. We found differences in search rates for the term "desomorphine" at different administrative scales. For example at the level of the 8 Russian federal regions, there was little differentiation in patterns of behaviour consistent with an interest in the production and use of krokodil. Similarly, within oblasts, we noted considerable variation between urban centres in the same oblast.

However, results from provincial cities should be regarded with caution. The absolute volume of search in smaller oblast cities can be low, and results should be interpreted with caution. For example, Kamensk-Uralskiy city in Sverdlovsk region, with a 2010 population of 174689 recorded a mean of 20.226 searches per month for the term "desomorphine" between December 2011 and May 2012. This suggested potentially high per capita rates of krokodil use during that period. However, this apparently high search rate equated to 35 searches per month. See figure 9. Our results suggest

investigation of Yandex data from all available urban centres in each oblast is recommended when investigating patterns of illicit drug use.
