# Supplementary figures and images for: Internet Search and Krokodil in the Russian Federation: An Infoveillance Study
Source: J Med Internet Res. 2014 Sep 18;16(9):e212. doi: 10.2196/jmir.3203 (PMC4180331; doi:10.2196/jmir.3203)

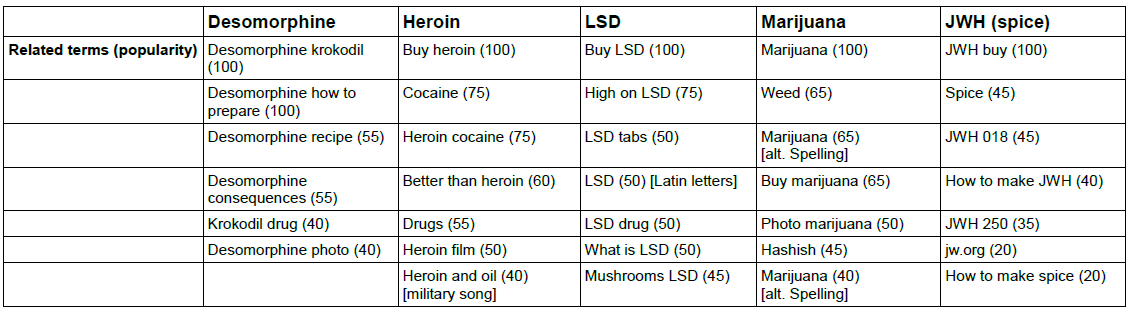

Supplement: Supplementary file 4 [file jmir_v16i9e212_app4.jpg]

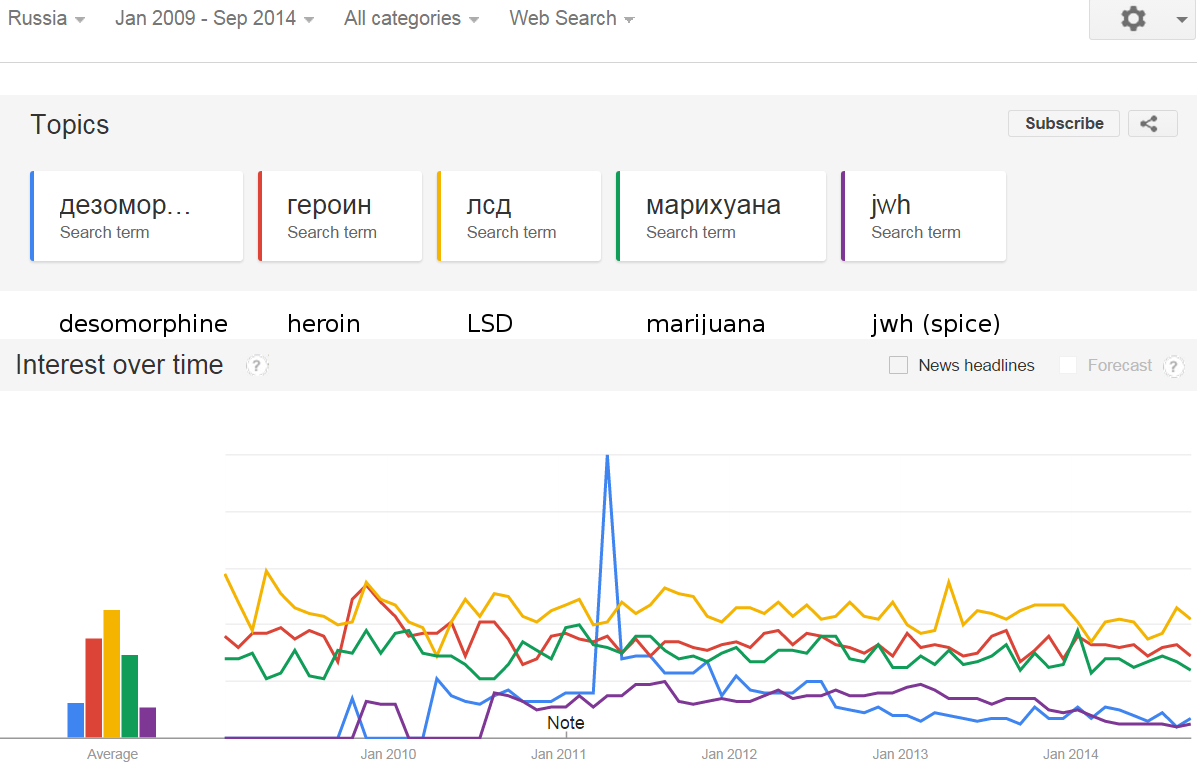

Supplement: Supplementary file 5 [file jmir_v16i9e212_app5.jpg]
